# Online public interest in common malignancies and cancer screening during the COVID-19 pandemic in the United States

**Published:** 2021-11-06

**Authors:** Samuel A. Cohen, Shayan Ebrahimian, Landon E. Cohen, Jonathan D. Tijerina

**Affiliations:** ^1^Stanford University School of Medicine, 291 Campus Drive, Stanford, CA, 94305, United States of America; ^2^UCLA David Geffen School of Medicine, 10833 Le Conte Avenue, Los Angeles, CA, 90095, United States of America; ^3^Keck School of Medicine of USC, 1975 Zonal Avenue, Los Angeles, CA, 90089, United States of America; ^4^Bascom Palmer Eye Institute, 900 NW 17^th^ Street, Miami, FL 33136, United States of America

**Keywords:** public interest, cancer, COVID-19, screening, online

## Abstract

**Background and Aim:**

The COVID-19 pandemic was declared a national emergency in the United States in March 2020. The Centers for Medicare and Medicaid Services subsequently released recommendations that health-care facilities temporarily delay elective surgeries and non-essential medical procedures. Disruptions to medical care significantly impacted cancer patients, with cancer screenings halted and nonurgent cancer surgeries postponed as health-care facilities shifted resources toward the COVID-19 pandemic. Although it has been reported that cancer screening rates decreased dramatically in the United States in 2020, it is unclear whether this trend was driven by factors related to public interest in cancer and/or cancer screening as opposed to other factors such as clinical backlogs, pandemic-related policies, and/or resource limitations. The purpose of this study was to use the Google Trends tool to evaluate public interest in six common malignancies and four common cancer screening methods during the COVID-19 pandemic.

**Methods:**

We used the Google Trends tool to quantify public interest in six different malignancies (Breast Cancer, Colon Cancer, Lung Cancer, Prostate Cancer, Thyroid Cancer, and Cervical Cancer) and four cancer screening methods (Pap Smear, Lung Cancer Screening, Mammogram, and Colonoscopy) in the United States during the COVID-19 pandemic. Welch’s t-tests were used to compare monthly search volumes during the COVID-19 pandemic (2020) to the 4 years before the pandemic (2016 – 2019) for all ten search terms included in our study. We used Benjamini-Hochberg to adjust raw p values to account for multiple statistical comparisons. The level of statistical significance was defined by choosing a false discovery rate of 0.05.

**Results:**

Our results indicate significantly reduced interest in all malignancies studied at the beginning of the COVID-19 pandemic. Public interest in [‘Breast Cancer’], [‘Colon Cancer’], [‘Lung Cancer’], [‘Thyroid Cancer’], and [‘Cervical Cancer’] significantly decreased in the months of March, April, May, and June 2020 when compared with public interest in 2016-2019. Public interest in cancer screening methods such as [‘Pap Smear’], [‘Lung Cancer Screening’], [‘Mammogram’], and [‘Colonoscopy’] significantly deceased in the months of April and May compared to 2016 – 2019 values. However, decreased public interest in cancer screening methods was temporary, with Google search volumes returning to pre-pandemic levels in June 2020 – December 2020.

**Conclusion:**

There was significantly reduced public interest in both common malignancies and cancer screening methods at the beginning of the COVID-19 pandemic in the United States. However, after an initial decline, public interest as indicated by Google search volumes quickly returned to pre-pandemic levels in the second half of the calendar year 2020. In addition, trends in public interest in cancer screening as indicated by Google search volumes aligned with cancer screening uptake rates in the United States during the study period. This finding suggests that Google Trends may serve as an effective tool in gauging the public’s interest in cancer and/or cancer screenings in the United States, which makes it a valuable resource that can be used to inform decisions aimed at improving cancer screening rates in the future.

**Relevance for Patients:**

The Google Trends tool can be used to measure public interest in various malignancies and their associated screening methods. Google Trends data may be used to inform measures aimed at improving cancer screening uptake.

## 1. Introduction

The COVID-19 pandemic has fundamentally changed the healthcare landscape for both healthcare professionals and patients. On March 13, 2020, the President of the United States declared COVID-19 to be a national emergency [1]. The Centers for Medicare and Medicaid Services (CMS) subsequently released recommendations that health-care facilities “[delay] all elective surgeries, non-essential medical, surgical, and dental procedures” [2]. The recommendations issued by CMS temporarily brought health-care systems to a halt as resources were shifted to focus on the COVID-19 pandemic [3]. Disruptions in healthcare services significantly impacted cancer patients, as routine cancer screening procedures were advised against and nonurgent cancer surgeries were delayed [4,5].

More than 1 year after the initial declaration of COVID-19 as a national emergency, concerns have been raised regarding the impact of the pandemic on cancer care, especially with regard to delays in diagnosis and treatment that may have occurred as a result of the pandemic [5]. For example, a recent study examining cancer screening rates in the United States revealed substantial decreases in cancer screenings, visits, and surgeries in 2020 when compared with metrics observed in the previous year’s [6]. It is unclear to what extent public apprehension regarding cancer screening during the COVID-19 pandemic was a factor in driving reduced cancer screening rates in 2020 when compared with other factors such as clinical backlogs, pandemic-related policies, and/or pandemic-related resource limitations [7-9]. As such, we sought to quantify public interest in various malignancies and cancer screening methods during the COVID-19 pandemic.

The internet is one way to track public interest in cancer-related topics [10]. When searching for cancer information online, the primary search engine that patients use is Google, which accounts for more than 90% of all internet searches [11]. Google Trends is a free, open-source tool that allows customizable analysis of search term volumes entered into the Google search engine. The Google Trends tool has been utilized previously to measure public interest in a wide range of health topics, from influenza outbreaks to osteoarthritis treatments to plastic surgery procedures [12-17]. In addition, the Google Trends tool has been used extensively to measure public interest in various oncological topics such as the effectiveness of cancer awareness months [18,19]. The purpose of this study was to use the Google Trends tool to evaluate public interest in six common malignancies and four common cancer screening methods during the COVID-19 pandemic. We hypothesized that there would be a reduction in public interest in common malignancies and cancer screening methods in the months after the onset of the pandemic in the United States. A sustained reduction in public awareness of cancer and cancer screening as a result of the pandemic has important clinical implications.

## 2. Methods

In this cross-sectional retrospective study, we used the Google Trends tool to investigate the impact of the COVID-19 pandemic on public interest on various malignancies and cancer screening methods in the United States.

### 2.1. Google trends output

Google Trends analyses can be customized by search term, time period, and geographic location. After a search term is entered into the Google Trends tool and temporal and geographic constraints are specified, Google Trends generates visuals and outputs that reflect the volume of a given search term relative to peak popularity within the defined time period, which is assigned a value of 100. The data are presented as relative search volume (RSV), which is computed as ratio between searches for a given topic and the total amount of Google queries. An RSV value of 100 indicates the largest ratio between searches for a specific topic and the total amount of Google queries, while an RSV of 0 indicates that at the specified time point, the proportion of queries for the search term was <1% of its peak RSV (RSV 100) [20]. The Google Trends tool uses RSV rather than absolute count of Google searches to allow for ease of comparison of search volumes in states, cities, and countries with varying population densities. In the current study, the following filters were utilized in the Google Trends tool: Search Term: [Malignancy or Cancer Screening Method of Interest], Time Period: [January 1, 2016 – December 31, 2020], Geographic Location: [United States].

### 2.2. Search term selection

We investigated trends in public interest for six common malignancies and four common cancer screening methods. The six malignancies investigated include the following: [‘Breast Cancer’], [Colon Cancer], [‘Lung Cancer’], [‘Prostate Cancer’], [‘Thyroid Cancer’], and [‘Cervical Cancer’]. The four cancer screening methods investigated include the following: [‘Pap Smear’], [‘Lung Cancer Screening’], [‘Mammogram’], and [‘Colonoscopy’]. The six aforementioned malignancies were chosen due to their high relative frequency and/or available screening methods. The specific search terms for the four cancer screening methods were selected as a result of their demonstrated popularity using the “Related Queries” feature of the Google Trends tool.

### 2.3. Statistical analysis

The Google Trends tool provided RSV on a weekly basis from 2016 to 2020 for all ten search terms (six malignancies and four cancer screening methods) included in this study. To account for monthly fluctuations in RSV during cancer awareness months, we compared monthly search volume data during the COVID-19 pandemic (March – December, 2020) to the same months in the prior 4 years (March – December, 2016 – 2019) for each of the ten search terms included in our study. The months of January and February were excluded from analysis due to the onset and declaration of the COVID-19 pandemic as a global health emergency in March 2020 [1]. Welch’s t-tests were used to compare monthly search volumes for 10 months (March – December) for all ten search terms included in our study, for a total of 100 t-tests. We used Benjamini-Hochberg (BH) to adjust raw p values to account for multiple statistical comparisons [21]. The adjusted BH p values were obtained using SPSS Version 26.0.0.1. Our significance level was determined by controlling the expected proportion of false discoveries at 0.05.

## 3. Results

**Figure 1:**
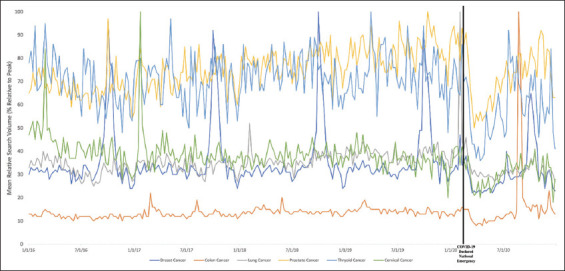
Google Trends relative search volumes (RSV) from January 2016 to December 2020 for [‘Breast Cancer’], [‘Colon Cancer’], [‘Lung Cancer’], [‘Prostate Cancer’], [‘Thyroid Cancer’], and [‘Cervical Cancer’].

The monthly RSV for the six malignancies studied are displayed in Figure 1. We compared the mean monthly RSV during the pandemic (March - December, 2020) to RSV prior to the pandemic (mean pooled values including data from 2016 to 2019), and the results are displayed in Tables 1-6. Raw p values and adjusted BH p values due to multiple comparisons are both listed.

**Table 1:** Relative search volumes for [‘Breast Cancer’] during the COVID-19 pandemic (2020), as compared with the prior 4 years (2016 – 2019)

Month	Mean RSV, 2016 – 2019	Mean RSV, 2020	Raw p value	BH-Adjusted p value
March	32.28	25.20	0.0131	**0.0354**
April	32.10	22.75	1.520E-12	**1.520E-10**
May	31.46	23.40	1.1289E-09	**3.763E-8**
June	30.78	26.25	1.182E-07	**1.689E-6**
July	31.25	31.50	0.9272	0.9658
August	31.31	29.20	0.0116	**0.0010**
September	48.38	39.75	0.3086	0.4171
October	64.79	53.25	0.1113	0.1890
November	31.81	28.40	0.0617	0.1210
December	29.38	27.00	0.3488	0.4530

RSV: Relative Search Volume; BH: Benjamini-Hochberg. BH-adjusted p values that are less than 0.05 are in bold.

**Table 2:** Relative search volumes for [‘Colon Cancer’] during the COVID-19 pandemic (2020), as compared with the prior 4 years (2016 – 2019)

Month	Mean RSV, 2016 – 2019	Mean RSV, 2020	Raw p value	BH-Adjusted p value
March	15.33	10.80	0.0165	**0.0413**
April	14.16	9.25	0.0002	**0.0012**
May	13.34	9.80	7.0911E-06	**5.909E-5**
June	12.99	11.50	0.0043	**0.0188**
July	13.28	13.00	0.6908	0.7676
August	13.44	41.60	0.1998	0.3027
September	12.71	17.50	0.0170	**0.0415**
October	13.45	16.00	0.0270	0.0614
November	12.75	14.00	0.2908	0.3984
December	12.44	16.50	0.1734	0.2753

RSV: Relative Search Volume; BH: Benjamini-Hochberg. BH-adjusted p values that are less than 0.05 are in bold.

**Table 3:** Relative search volumes for [‘Lung Cancer’] during the COVID-19 pandemic (2020), as compared with the prior 4 years (2016 – 2019)

Month	Mean RSV, 2016 – 2019	Mean RSV, 2020	Raw p value	BH-Adjusted p value
March	36.80	32.40	0.0104	**0.0316**
April	36.35	29.75	9.795E-07	**9.795E-6**
May	35.18	28.80	5.391E10-7	**5.990E-6**
June	33.81	29.25	0.0002	**0.0010**
July	33.19	31.50	0.1542	0.2488
August	31.88	31.00	0.5195	0.6185
September	34.90	32.00	0.0075	**0.0270**
October	36.21	35.75	0.9397	0.9687
November	36.19	30.20	0.0079	**0.0262**
December	34.23	30.25	0.0269	0.0626

RSV: Relative Search Volume; BH: Benjamini-Hochberg. BH-adjusted p values that are less than 0.05 are in bold.

**Table 4:** Relative search volumes for [‘Prostate Cancer’] during the COVID-19 pandemic (2020), as compared with the prior 4 years (2016 – 2019)

Month	Mean RSV, 2016 – 2019	Mean RSV, 2020	Raw p value	BH-Adjusted p value
March	75.05	62.80	0.0736	0.1314
April	74.91	54.50	2.937E-9	**7.343E-8**
May	70.98	60.40	0.0007	**0.0037**
June	74.89	69.50	0.0357	0.0761
July	74.39	72.50	0.7557	0.8215
August	74.94	77.00	0.4804	0.5931
September	75.66	74.75	0.5523	0.6423
October	82.41	79.25	0.5020	0.6049
November	75.56	85.00	0.0701	0.1279
December	71.26	70.25	0.7821	0.8320

RSV: Relative Search Volume; BH: Benjamini Hochberg. BH-adjusted p values that are less than 0.05 are in bold.

**Table 5:** Relative search volumes for [‘Thyroid Cancer’] during the COVID-19 pandemic (2020), as compared with the prior 4 years (2016 – 2019)

Month	Mean RSV, 2016 – 2019	Mean RSV, 2020	Raw p value	BH-Adjusted p value
March	74.68	48.40	0.0053	**0.0204**
April	74.06	38.50	7.245E-11	**3.623E-9**
May	74.94	49.80	1.245E-6	**1.132E-5**
June	71.54	58.00	0.0043	**0.0188**
July	71.33	66.25	0.6797	0.7637
August	71.19	72.60	0.7605	0.8177
September	70.86	59.50	0.0057	**0.0212**
October	71.93	67.75	0.3868	0.4896
November	68.88	57.20	0.0511	0.1043
December	63.85	57.25	0.5393	0.6345

RSV: Relative Search Volume; BH: Benjamini-Hochberg. BH-adjusted p values that are less than 0.05 are in bold.

**Table 6:** Relative search volumes for [‘Cervical Cancer’] during the COVID-19 pandemic (2020), as compared with the prior 4 years (2016 – 2019)

Month	Mean RSV, 2016 – 2019	Mean RSV, 2020	Raw p value	BH-Adjusted p value
March	40.71	27.20	0.0113	**0.0323**
April	39.13	24.75	0.0008	**0.0042**
May	37.46	25.20	9.062E-6	**6.971E-5**
June	38.69	29.75	0.0015	**0.0073**
July	38.65	35.5	0.2215	0.3210
August	37.56	34.00	0.0661	0.1248
September	38.05	32.25	0.0147	**0.0377**
October	37.94	35.75	0.3386	0.4455
November	37.69	32.20	0.1158	0.1930
December	33.78	28.00	0.1848	0.2890

RSV: Relative Search Volume; BH: Benjamini-Hochberg. BH-adjusted p values that are less than 0.05 are in bold.

With regard to public interest in [‘Breast Cancer’], a statistically significant reduction in RSV, adjusted for multiple comparisons, at a significance cutoff of p < 0.05 was observed in 2020 in the months of March, April, May, June, and August. The largest drop in public interest (9.4% decrease in mean RSV compared with 2016 – 2019) was observed in April (Table 1).

For [‘Colon Cancer’], a significant reduction in RSV in 2020 was observed in the months of March, April, May, and June. The largest drop in public interest (4.9% decrease in mean RSV compared with 2016-2019) was observed in April. There was a significant increase in public interest in [‘Colon Cancer’] in September 2020 when compared with September 2016 – 2019 (Table 2).

For [‘Lung Cancer’], a significant reduction in RSV in 2020 was observed in the months of March, April, May, June, September, and November. The largest drop in public interest (6.6% decrease in mean RSV compared with 2016-2019) was observed in April (Table 3).

For [‘Prostate Cancer’], a significant reduction in RSV in 2020 was observed in the months of April and May. The largest drop in public interest (20.4% decrease in mean RSV compared with 2016 – 2019) was observed in April.

For [‘Thyroid Cancer’], a significant reduction in RSV in 2020 was observed in the months of March, April, May, June, and September. The largest drop in public interested (35.6% decrease in mean RSV compared with 2016 – 2019) was observed in April (Table 5).

**Figure 2:**
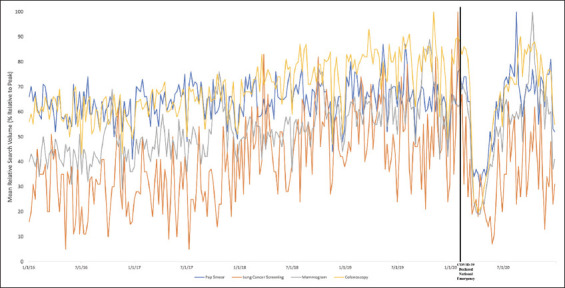
Google Trends relative search volumes (RSV) from January 2016 to December 2020 for [‘Pap Smear’], [‘Lung Cancer Screening’], [‘Mammogram’], and [‘Colonoscopy’].

The monthly RSV for the four cancer screening methods studied are displayed in Figure 2. Comparisons of monthly RSV in the 4 years before the pandemic (2016 – 2019) to those observed during the COVID-19 pandemic (2020) is observed in Tables 7-10.

**Table 7:** Relative search volumes for [‘Pap Smear’] during the COVID-19 pandemic (2020), as compared with the prior 4 years (2016 – 2019)

Month	Mean RSV, 2016 – 2019	Mean RSV, 2020	Raw p value	BH-Adjusted p value
March	61.74	47.60	0.1040	0.1825
April	62.73	34.25	1.846E-7	**2.308E-6**
May	65.55	44.20	0.0005	**0.0025**
June	67.88	62.00	0.0670	0.1241
July	68.06	74.00	0.0369	0.0769
August	66.19	77.40	0.1253	0.2055
September	62.08	65.75	0.3295	0.4393
October	63.86	71.50	0.0235	0.0559
November	61.38	67.60	0.2019	0.3012
December	57.84	65.25	0.4123	0.5154

RSV: Relative Search Volume; BH: Benjamini Hochberg. BH-adjusted p values that are less than 0.05 are in bold.

**Table 8:** Relative search volumes for [‘Lung Cancer Screening’] during the COVID-19 pandemic (2020), as compared with the prior 4 years (2016 – 2019)

Month	Mean RSV, 2016 – 2019	Mean RSV, 2020	Raw p value	BH-Adjusted p value
March	43.06	26.60	0.0115	**0.0321**
April	42.68	25.50	0.0083	**0.0277**
May	38.88	13.40	4.870E-5	**0.00003**
June	31.50	31.50	0.9520	0.9715
July	34.65	43.50	0.2683	0.3779
August	42.13	39.20	0.7249	0.7966
September	42.31	41.25	0.8065	0.8490
October	37.25	50.50	0.0328	0.0714
November	44.81	33.80	0.2690	0.3738
December	34.86	33.00	0.6765	0.7687

RSV: Relative Search Volume; BH: Benjamini-Hochberg. BH-adjusted p values that are less than 0.05 are in bold.

**Table 9:** Relative search volumes for [‘Mammogram’] during the COVID-19 pandemic (2020), as compared with the prior 4 years (2016 – 2019)

Month	Mean RSV, 2016 – 2019	Mean RSV, 2020	Raw p value	BH-Adjusted p value
March	50.18	38.60	0.2028	0.2983
April	48.49	21.25	9.202E-9	**1.840E-7**
May	48.68	37.20	0.0087	**0.0280**
June	52.34	50.50	0.4857	0.5923
July	49.71	60.25	0.0043	**0.0197**
August	50.94	59.20	0.0045	**0.0181**
September	54.33	63.75	0.2221	0.3174
October	69.91	85.75	0.0632	0.1215
November	54.81	63.40	0.0601	0.1210
December	48.66	49.25	0.9890	0.9891

RSV: Relative Search Volume; BH: Benjamini Hochberg. BH-adjusted p values that are less than 0.05 are in bold.

**Table 10:** Relative search volumes for [‘Colonoscopy’] during the COVID-19 pandemic (2020), as compared with the prior 4 years (2016 – 2019)

Month	Mean RSV, 2016 – 2019	Mean RSV, 2020	Raw p value	BH-Adjusted p value
March	72.13	48.00	0.1068	0.1842
April	70.45	23.50	3.555E-8	**7.343E-8**
May	65.25	45.20	0.0134	**0.0352**
June	70.99	61.50	0.0304	0.0675
July	71.11	70.50	0.9682	0.9779
August	71.00	78.20	0.1959	0.3013
September	68.84	82.25	0.0104	**0.0325**
October	71.74	85.75	1.147E-5	**8.190E-5**
November	69.13	73.60	0.3825	0.4904
December	62.59	67.00	0.5584	0.6418

RSV: Relative Search Volume; BH: Benjamini-Hochberg. BH-adjusted p values that are less than 0.05 are in bold.

For [‘Pap Smear’], a statistically significant reduction in RSV, adjusted for multiple comparisons, at a significance cutoff of p < 0.05 was observed in 2020 in the months of April and May. The largest drop in public interested (28.5% decrease in mean RSV compared with 2016 – 2019) was observed in April (Table 7).

For [‘Lung Cancer Screening’], a significant reduction in RSV in 2020 was observed in the months of March, April and May. The largest drop in public interested (25.5% decrease in mean RSV compared with 2016 – 2019) was observed in April (Table 8).

For [‘Mammogram’], a significant reduction in RSV in 2020 was observed in the months of April and May. The largest drop in public interested (25.5% decrease in mean RSV compared with 2016 – 2019) was observed in April. There was a significant increase in public interest in [‘Mammogram’] in July and August 2020 when compared with July and August 2016 – 2019, respectively (Table 9).

For [‘Colonoscopy’], a significant reduction in RSV in 2020 was observed in the months of April and May. The largest drop in public interested (47.0% decrease in mean RSV compared with 2016 – 2019) was observed in April (Table 10). There was a significant increase in public interest in [‘Colonoscopy’] in September and October 2020 when compared with September and October 2016 – 2019, respectively (Table 10).

## 4. Discussion

The purpose of this study was to examine the impact of the COVID-19 pandemic on public interest in various malignancies and cancer screening methods in the United States. Our results indicate significantly reduced interest in all six cancers studied for many of the months included in our study. Public interest in [‘Breast Cancer’], [‘Colon Cancer’], [‘Lung Cancer’], [‘Thyroid Cancer’], and [‘Cervical Cancer’] significantly decreased in the year 2020 in the months of March, April, May, and June when compared with public interest in 2016-2019. Public interest in [‘Prostate Cancer’] significantly decreased in April and May, 2020, when compared with 2016 – 2019 values. For some cancers, such as [‘Breast Cancer’], [‘Lung Cancer’], [‘Thyroid Cancer’], and [‘Cervical Cancer’], a decrease in public interest endured slightly longer, with significant reductions in public interest in August and/or September. With regards to public interest in cancer screening methods, our results indicate that public interest in [‘Pap Smear’], [‘Lung Cancer Screening’], [‘Mammogram’], and [‘Colonoscopy’] significantly deceased in the year 2020 in the months of April and May when compared with public interest in 2016 – 2019. However, the decrease in public interest observed in the cancer screening methods studied was limited to the months in the beginning of the COVID-19 pandemic, with search volumes for [‘Pap Smear’], [‘Lung Cancer Screening’], [‘Mammogram’], and [‘Colonoscopy’] returning to pre-pandemic levels (or even exceeding previous levels) observed in 2016 – 2019 in the months of June-December.

Public interest in various malignancies as indicated by online search activity has previously been correlated with incidence and mortality rates of some cancers [22,23]. In addition, the Google Trends tool has been shown to be a valuable tool for analyzing public interest in cancer screening in several different countries [24,25]. As such, the results of our study have clinical implications, with the potential to inform patient counseling. Our findings align with the conclusions of a recently published study examining the effect of the COVID-19 pandemic on public interest in various cancers in Canada. In both the United States and Canada, public interest in many of the most common malignancies decreased significantly during the first few of the months of the pandemic before normalizing toward the end of the calendar year 2020 [26].

In addition to analyzing public interest in many common cancers, we also examined trends in public interest in various cancer screening methods. While public interest in all search terms associated with cancer screening decreased significantly in the first few months of the pandemic, search volumes largely normalized to pre-pandemic levels by the end of the calendar year 2020. The results of our study align with a recently published article that examined breast, colorectal, and prostate cancer screening rates in the United States. Chen *et al*. [4] reported a sharp decrease in cancer screening uptake in the United States in the months of March through May, which is the same trend that we observed with regards to public interest in various cancer screening methods using the Google Trends tool during the same time period.

The result of our study has implications for policy makers and nonprofit organizations tasked with improving cancer screening rates in the United States. Near the beginning of the pandemic, nonemergent medical services such as cancer screenings drastically decreased [4]. In addition to the frequency of nonemergency medical services declining, there was also a sharp reduction in emergency department visits; with many people reporting that they felt uncomfortable visiting the emergency department because of a fear of becoming infected with COVID-19 [27]. This led to many patients waiting to seek treatment for life-threatening conditions, which led to adverse health consequences [28,29]. As a result, many hospitals launched initiatives aimed at easing patients’ fears about the virus and encouraged them to continue their preventative care regimens, such as cancer screenings, that are vital to their overall health [27,30]. The messaging regarding the importance of attending to cancer screenings was well received, as supported by our findings of increased public interest in common malignancies and cancer screening methods throughout the rest of the 2020 calendar year. However, despite revived public interest in cancer screening, reduced screening totals for the remainder of calendar year 2020 when compared with the previous year’s suggest that resources focused on improving cancer rates should be dedicated to other factors such as clinical backlogs, pandemic-related legislation, and health-care center resource limitations that appear to be decreasing screening uptake, [4,7-9,31]. At this time, properly dedicating resources to these areas of focus, rather than focusing on messaging aimed at easing concerns of the public, could help to improve cancer screening rates in the future.

The results of our study also provide insight into how public interest patterns in various malignancies and cancer screening methods vary due to media coverage. Previously published reports indicate that public awareness campaign and celebrity diagnoses or deaths significantly impact the public’s interest in various malignancies [18,32]. We observed similar trends in our study. When comparing public interest in cancers or cancer screening methods in 2020 to public interest in 2016-2019, there were limited circumstances (6 months out of 100 months studied) in which the 2020 search volumes were greater than those observed in 2016-2019. However, in each of the six circumstances, greater public interest in 2020 was driven by a media-generating event such as a celebrity diagnosis/death or a cancer awareness month. For example, in late August of 2020, renewed actor Chadwick Boseman died of colon cancer. There was a subsequent increase in public interest in both [‘Colon Cancer’] and [‘Colonoscopy’] in September and/or October after the news was shared by media outlets around the world (Tables 2 and 10). Health-care organizations tasked with improving cancer screening rates and outreach initiatives should utilize the tremendous publicity generated by a celebrity cancer diagnoses or death to raise awareness and to provide important information about cancer screening to the public, which may help to decrease related mortality rates.

One long-lasting effect of the COVID-19 pandemic may be a shift in the way in which people expect to undergo their cancer screenings. The convenience of the in-home cancer screening test has become even more valuable to patients who are hesitant to leave their homes during the pandemic, and the race is underway to develop the most accurate in-home screening methods. Stool-based screening tests that patients perform at home in an effort to detect colon cancer gained popularity over the pandemic and are expected to continue to serve as an efficient alternative to the colonoscopy in the upcoming years [33,34]. In addition, self-sampling kits for cervical cancer screening are currently under evaluation for approval by the United States Food and Drug Association [35]. It is possible that the convenience of an in-home screening method may improve screening rates, particularly among people who feel uncomfortable with the screening methods that are used in a traditional healthcare setting for breast, cervical, and colorectal cancer screening [36-38]. In addition, increased uptake of in-home screening methods could help to avoid a steep decline in cancer screening that would likely occur in the event of a future pandemic. As such, it would be beneficial to use the Google Trends tool to monitor the public’s interest with regards to in-home cancer screening tests in the future.

The findings of our study suggest that Google Trends can serve as an effective tool in gauging the public’s interest in cancer screenings in the United States. The patterns observed with regards to public interest in various cancer screenings such as [‘Pap Smear’], [‘Lung Cancer Screening’], [‘Mammogram’], and [‘Colonoscopy’] largely mimic those seen in actual health-care screening uptake in the United States - a sharp decline at the beginning of the pandemic followed by a gradual increase throughout the remainder of the calendar year 2020 [39]. In addition to the long-term benefits of a free, open-source tool that can track public interest in cancer screening, the data provided by the Google Trends tool may prove critical should virus variants that are resistant to modern vaccines emerge and extend the duration of the COVID-19 pandemic. The rise of the “delta variant” of the virus has coincided with an increase in breakthrough infections and the potential of a return to more restrictive measures in the United States [40]. In the event of restrictions being implemented in the future similar to those that were implemented at the beginning of the COVID-19 pandemic, the Google Trends tool can be utilized by healthcare systems to provide real-time, up to date data regarding public interest in cancer screenings, which can contribute to proper resource allocation to meet the screening demands of a given geographical area.

There are several limitations to this study. First, casual inferences cannot be drawn due to the observational nature of the study design. In addition, Google Trends does not provide extensive information about the demographics of the users whose data are reflected in this study. As such, it is unclear if the users are a representative sample of the United States population. Next, the Google Trends tool only captures information about searches that are entered into the Google search engine. People may seek information about various malignancies and cancer screening methods using other search engines, and this data would not be reflected in this study. However, more than 90% of search engine inquiries worldwide are executed using Google as compared to an alternative search engine, which supports the notion that the data included in our study is representative of all queries in the United States [11]. Finally, due to the de-identified nature of the Google users whose data are reflected in this study, we cannot directly link public interest in various cancer screening methods with actual cancer screening uptake. However, the trends in public interest in cancer screening methods observed in our study mimic the trends of cancer screening uptake observed in recently published studies, which suggests that the Google Trends tool may be an effective gauge for public interest in cancer screening [4,39].

## 5. Conclusions

Google search trends indicate decreased public interest in many common malignancies and cancer screening methods in the United States in the early months of the COVID-19 pandemic, with a gradual return to pre-pandemic levels towards the end of the 2020 calendar year. Furthermore, trends in public interest in several cancer screening methods as indicated by Google search volumes aligned with cancer screening uptake in the United States, suggesting that the Google Trends tool may be a valuable information source that can be used to guide decisions aimed at improving cancer screening rates in the future. Finally, we observed the strong impact that the media can have on driving public interest in various malignancies and cancer screening methods. Our findings may be useful to organizations attempting to educate the public regarding various malignancies with the primary goal of improving cancer screening uptake in the United States during the COVID-19 pandemic and into the future.

### Conflict of Interest

The authors declare no conflicts of interest.
